# Boron Deficiency Increases Cytosolic Ca^2+^ Levels Mainly via Ca^2+^ Influx from the Apoplast in *Arabidopsis thaliana* Roots

**DOI:** 10.3390/ijms20092297

**Published:** 2019-05-09

**Authors:** Carlos Quiles-Pando, M. Teresa Navarro-Gochicoa, M. Begoña Herrera-Rodríguez, Juan J. Camacho-Cristóbal, Agustín González-Fontes, Jesús Rexach

**Affiliations:** Departamento de Fisiología, Anatomía y Biología Celular, Universidad Pablo de Olavide, E-41013 Sevilla, Spain; cquipan@upo.es (C.Q.-P.); mtnavgoc@upo.es (M.T.N.-G.); mbherrod@upo.es (M.B.H.-R.); jjcamcri@upo.es (J.J.C.-C.); agonfon@upo.es (A.G.-F.)

**Keywords:** apoplastic calcium, boron deficiency, calcium signaling, cytosolic calcium, Cameleon YC3.6, *Arabidopsis thaliana*

## Abstract

Boron (B) is a micronutrient for plant development, and its deficiency alters many physiological processes. However, the current knowledge on how plants are able to sense the B-starvation signal is still very limited. Recently, it has been reported that B deprivation induces an increase in cytosolic calcium concentration ([Ca^2+^]_cyt_) in *Arabidopsis thaliana* roots. The aim of this work was to research in *Arabidopsis* whether [Ca^2+^]_cyt_ is restored to initial levels when B is resupplied and elucidate whether apoplastic Ca^2+^ is the major source for B-deficiency-induced rise in [Ca^2+^]_cyt_. The use of chemical compounds affecting Ca^2+^ homeostasis showed that the rise in root [Ca^2+^]_cyt_ induced by B deficiency was predominantly owed to Ca^2+^ influx from the apoplast through plasma membrane Ca^2+^ channels in an IP_3_-independent manner. Furthermore, B resupply restored the root [Ca^2+^]_cyt_. Interestingly, expression levels of genes encoding Ca^2+^ transporters (*ACA10*, plasma membrane P_IIB_-type Ca^2+^-ATPase; and *CAX3*, vacuolar cation/proton exchanger) were upregulated by ethylene glycol tetraacetic acid (EGTA) and abscisic acid (ABA). The results pointed out that ACA10, and especially CAX3, would play a major role in the restoration of Ca^2+^ homeostasis after 24 h of B deficiency.

## 1. Introduction

Plant ability to respond appropriately to variations in soil nutrient concentrations is of essential relevance for plant survival. Nutrients such as nitrate, phosphate, potassium, sulfate, and iron act as signals that can be perceived by plants [1]. Thus, in vascular plants, complex signaling pathways have evolved to sense their nutrient availability and, consequently, trigger a response that allows them to adapt to a changing environment [2]. Calcium (Ca^2+^) is likely the best-known second messenger that plays a major role in plant responses to diverse stresses and nutrient availability. Multiple stimuli induce specific spatio-temporal changes in cytosolic Ca^2+^ levels ([Ca^2+^]_cyt_), termed as “Ca^2+^ signatures” [3]. The precise shape of Ca^2+^ signatures is generated by Ca^2+^ movements between cytosol and specific cellular compartments, such as apoplasts, vacuoles (where Ca^2+^ concentration can reach mM values [4]), and the endoplasmic reticulum, through several Ca^2+^ channels and transporters. Ca^2+^ channels allow Ca^2+^ influx into the cytosol, while Ca^2+^ transporters are involved in Ca^2+^ efflux into particular reservoirs and apoplasts [3,5,6,7]. Ca^2+^ influx is performed by several categories of Ca^2+^-permeable channels, cyclic nucleotide-gated ion channels (CNGCs) being one of these types [8]. Although most of the CNGCs are localized in the plasma membrane [3,9,10], CNGC19 localizes to the tonoplast in *Arabidopsis* [11]. However, Ca^2+^-ATPase (ACAs) and Ca^2+^/H^+^ antiporters (CAXs) are the two main types of Ca^2+^ efflux systems that transport Ca^2+^ out of the cytosol, either to the apoplast or to intracellular reservoirs, against its electrochemical potential gradient [12].

Boron (B) is an essential element for plant development [13]. Its soil availability is an important factor that limits crop productivity and quality in different regions of the world [14,15]. Boron deprivation has been reported in nearly 90 countries affecting more than 100 plant species [16]. In fact, B availability causes important alterations in root and shoot growth at both vegetative and reproductive stages [17,18]. However, mechanisms through which B is involved in these developmental processes are not well-known. Nevertheless, the main function of this micronutrient is its structural role in the cell wall where borate forms a diester bond between apiose residues of two rhamnogalacturonan II monomers, providing an enhanced firmness to the cell wall [19,20]. Moreover, B deprivation does not only affect the cell wall but also disturbs many metabolic and physiological processes such as membrane and cytoskeleton structure and function, oxidative stress and secondary metabolism, nitrogen assimilation, and gene expressions, among others [14,18,21,22,23,24,25,26,27,28].

An issue of increasing interest is how plants sense B availability. Ca^2+^ has been involved in signaling process associated with the sensing of B deficiency by plants. BY-2 tobacco cells subjected to short-term B deprivation showed an increased Ca^2+^ uptake, likely via mechanosensitive Ca^2+^ channels [29]. In addition, B starvation enhanced [Ca^2+^]_cyt_ as well as the expression of Ca^2+^-related genes such as *CNGC19* (Ca^2+^ channel), several *ACAs* (Ca^2+^-ATPases), *CAX3* (Ca^2+^/H^+^ antiporter), various *CMLs* (calmodulin-like proteins), and *CDPKs* (Ca^2+^-dependent protein kinases) in *Arabidopsis* roots [30]. Furthermore, very recently it has been reported that B deficiency enlarged [Ca^2+^]_cyt_ in the *Malus domestica* pollen tube tip [31]. Although these findings suggest that Ca^2+^ is involved in a signaling pathway triggered by B deficiency, currently, precise mechanisms underlying this route remain unknown. Therefore, the aim of this work was to analyze whether a B resupply provokes a restoration of [Ca^2+^]_cyt_, and elucidate whether the rise in [Ca^2+^]_cyt_ triggered by B deprivation is due to a Ca^2+^ influx from the extracellular medium or from intracellular Ca^2+^ reservoirs. For these purposes, in vivo fluorescence measurements of [Ca^2+^]_cyt_ in *Arabidopsis* seedlings subjected to B starvation and, subsequently, resupply experiments were performed. In addition, [Ca^2+^]_cyt_ was determined in the presence of several chemical agents known to affect calcium homeostasis.

## 2. Results and Discussion

### 2.1. Cytosolic Calcium Levels Are Restored When Boron (B) Is Resupplied

**Figure 1:**
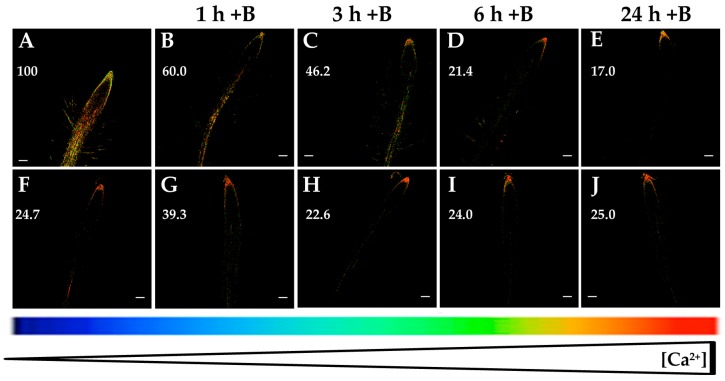
Fluorescence images of roots from *Arabidopsis* seedlings expressing the fluorescence resonance energy transfer (FRET)-based Ca^2+^ sensor UbiQ10:YC3.6-bar#22-2. Seedlings were subjected to boron (B) deprivation for 24 h (**A**) and, subsequently, they were transferred to media supplemented with 2 µM B for 1 h (**B**), 3 h (**C**), 6 h (**D**), and 24 h (**E**). In addition, seedlings grown with 2 µM B (**F**) were transferred to the same media (2 µM B) for 1 h (**G**), 3 h (**H**), 6 h (**I**), and 24 h (**J**), as a control. Fluorescence was monitored using settings for cpVenus excitation and emission. Increase in the FRET reflects higher [Ca^2+^]_cyt_ levels. For more details see Materials and Methods. Representative images: (**A**) *n* = 12 roots; (**B**) *n* = 5 roots; (**C**) *n* = 5 roots; (**D**) *n* = 7 roots; (**E**) *n* = 8 roots; (**F**) *n* = 12 roots; (**G**) *n* = 4 roots; (**H**) *n* = 4 roots; (**I**) *n* = 4 roots; and (**J**) *n* = 4 roots. Data are from a representative experiment that was repeated twice with very similar results. Scale bars represent 100 µm. Numbers indicate raw integrated density (%), obtained from ImageJ software, compared to the maximum fluorescence level (Figure 1A).

It was described that B starvation induced overexpression of stress-responsive genes in tobacco BY-2 cells and a higher Ca^2+^ influx when compared to control cells [29]. These results were consistent with the increased root [Ca^2+^]_cyt_ and expression of Ca^2+^-related genes described in *Arabidopsis* plants upon 6 and 24 h of B deficiency [30]. With the aim to analyze whether B resupply can restore [Ca^2+^]_cyt_ to initial levels prior to B starvation stimulus, *Arabidopsis* seedlings expressing YC3.6 were subjected to B deprivation for 24 h and, subsequently, were grown with 2 µM B for 1, 3, 6, or 24 h. At indicated times, fluorescence measurements were performed in *Arabidopsis* roots. Interestingly, a gradual decrease in fluorescence signal and, hence, in [Ca^2+^]_cyt_ was observed when plants were resupplied with 2 µM B (Figure 1A–E). However, when seedlings were maintained with 2 µM B, no significant changes in fluorescence levels were detected (Figure 1F–J). These data support not only that B deficiency rose [Ca^2+^]_cyt_ (Figure 1A,F; [30]), but also that this effect could be reversed by B resupply. Taken together, these findings suggested that root [Ca^2+^]_cyt_ was a significant parameter for the signaling of B deprivation.

**Figure 2:**
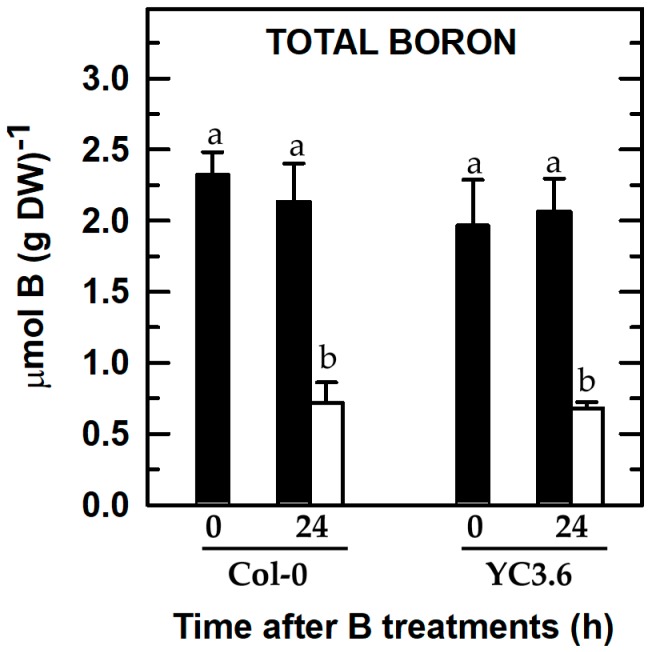
Total B concentration of roots from *Arabidopsis* Col-0 wild type and seedlings expressing the FRET-based Ca^2+^ sensor UbiQ10:YC3.6-bar#22-2. Seedlings were subjected (open bars) or not (filled bars) to B deprivation for 24 h. For more details see Materials and Methods. The results are given as means ± SD (*n* = 4 separate pools). Different letters have been used to designate statistically significant differences between Col-0 and/or YC3.6 seedlings subjected or not to B deficiency. Statistical analyses were performed according to ANOVA with Tukey’s HSD test (*p* < 0.05).

To ascertain whether root B concentration in the *Arabidopsis* Col-0 wild type and the line expressing Yellow Cameleon 3.6 (YC3.6) could be differently affected by B deficiency, total root B contents were determined. There was a remarkable decrease in the root B concentration in Col-0 wild type and seedlings expressing YC3.6 after 24 h of B deprivation (Figure 2). Furthermore, both lines had similar root B contents in the two B treatments, so that there were no statistically significant differences compared to each B treatment (Figure 2). These results supported that the findings shown in Figure 1 were regulated by B availability.

### 2.2. Apoplastic Calcium Is the Major Source for the Rise in Cytosolic Calcium Levels Induced by B Deficiency

It is widely known that temporal increases in [Ca^2+^]_cyt_ are performed through the calcium influx from several cellular compartments such as the vacuole, endoplasmic reticulum, and also from the apoplast [5,6]. These changes in [Ca^2+^]_cyt_ have been observed in response to a wide variety of abiotic stresses [5,6,32].

**Figure 3:**
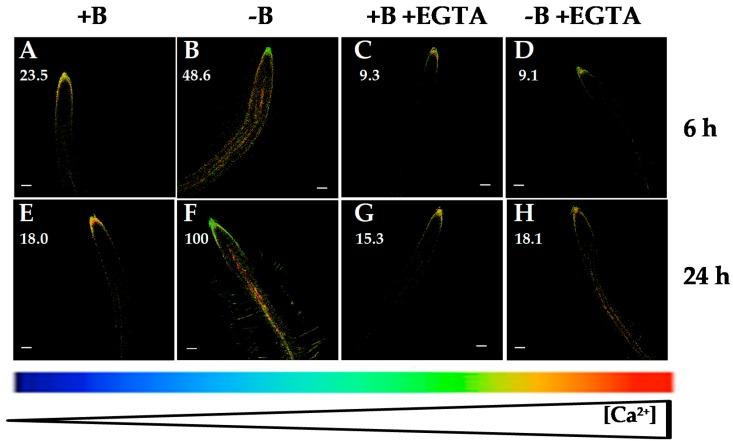
Fluorescence images of roots from *Arabidopsis* seedlings expressing the FRET-based Ca^2+^ sensor UbiQ10:YC3.6-bar#22-2 and treated or not with 1 mM ethylene glycol tetraacetic acid (EGTA). Seedlings were subjected (**B**,**D**,**F**,**H**) or not (**A**,**C**,**E**,**G**) to B deficiency for 6 (**A**–**D**) or 24 h (**E**–**H**) in the absence (**A**,**B**,**E**,**F**) or presence (**C**,**D**,**G**,**H**) of 1 mM EGTA. Fluorescence was monitored using settings for cpVenus excitation and emission. Increase in the FRET reflects higher [Ca^2+^]_cyt_ levels. For more details see Materials and Methods. Representative images: (**A**) *n* = 12 roots; (**B**) *n* = 12 roots; (**C**) *n* = 5 roots; (**D**) *n* = 6 roots; (**E**) *n* = 12 roots; (**F**) *n* = 12 roots; (**G**) *n* = 5 roots; and (**H**) *n* = 6 roots. Data are from a representative experiment that was repeated twice with very similar results. Scale bars represent 100 µm. Numbers indicate raw integrated density (%), obtained from ImageJ software, compared to the maximum fluorescence level (Figure 3F).

**Figure 4:**
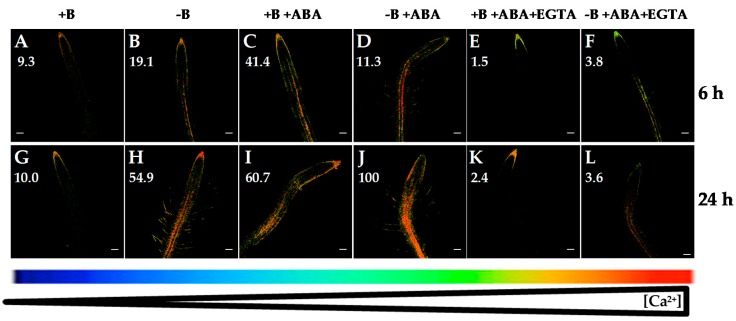
Fluorescence images of roots from *Arabidopsis* seedlings expressing the FRET-based Ca^2+^ sensor UbiQ10:YC3.6-bar#22-2 and treated or not with 5 µM ABA and/or 1 mM EGTA. Seedlings were subjected (**B**,**D**,**F**,**H**,**J**,**L**) or not (**A**,**C**,**E**,**G**,**I**,**K**) to B deficiency for 6 (**A**–**F**) or 24 h (**G**–**L**) in the absence (**A**,**B**,**G**,**H**) or presence (**C**,**D**,**I**,**J**) of 5 µM abscisic acid (ABA). In addition, B-sufficient and B-deficient seedlings were treated simultaneously with 5 µM ABA and 1 mM EGTA (**E**,**F**,**K**,**L**). Fluorescence was monitored using settings for cpVenus excitation and emission. Increase in the FRET reflects higher [Ca^2+^]_cyt_ levels. For more details see Materials and Methods. Representative images: (**A**) *n* = 12 roots; (**B**) *n* = 12 roots; (**C**) *n* = 7 roots; (**D**) *n* = 9 roots; (**E**) *n* = 9 roots; (**F**) *n* = 10 roots; (**G**) *n* = 12 roots; (**H**) *n* = 12 roots; (**I**) *n* = 10 roots; (**J**) *n* = 10 roots; (**K**) *n* = 9 roots; and (**L**) *n* = 8 roots. Data are from a representative experiment that was repeated twice with very similar results. Scale bars represent 100 µm. Numbers indicate raw integrated density (%), obtained from ImageJ software, compared to the maximum fluorescence level (Figure 4J).

**Figure 5:**
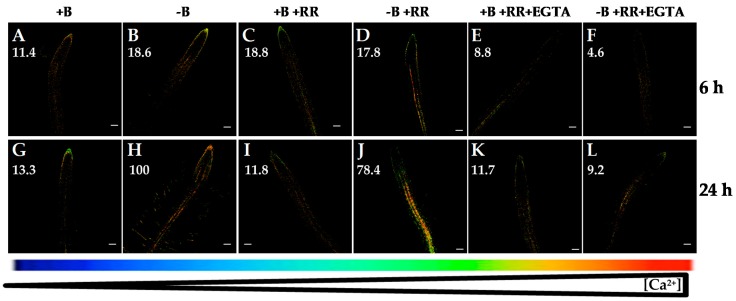
Fluorescence images of roots from *Arabidopsis* seedlings expressing the FRET-based Ca^2+^ sensor UbiQ10:YC3.6-bar#22-2 and treated or not with 50 µM ruthenium red (RR) and/or 1 mM EGTA. Seedlings were subjected (**B**,**D**,**F**,**H**,**J**,**L**) or not (**A**,**C**,**E**,**G**,**I**,**K**) to B deficiency for 6 (**A**–**F**) or 24 h (**G**–**L**) in the absence (**A**,**B**,**G**,**H**) or presence (**C**,**D**,**I**,**J**) of 50 µM RR. In addition, B-sufficient and B-deficient seedlings were treated simultaneously with 50 µM RR and 1 mM EGTA (**E**,**F**,**K**,**L**). Fluorescence was monitored using settings for cpVenus excitation and emission. Increase in the FRET reflects higher [Ca^2+^]_cyt_ levels. For more details see Materials and Methods. Representative images: (**A**) *n* = 12 roots; (**B**) *n* = 12 roots; (**C**) *n* = 9 roots; (**D**) n = 8 roots; (**E**) *n* = 9 roots; (**F**) *n* = 8 roots; (**G**) *n* = 12 roots; (**H**) *n* = 12 roots; (**I**) *n* = 7 roots; (**J**) *n* = 9 roots; (**K**) *n* = 8 roots; and (**L**) *n* = 7 roots. Data are from a representative experiment that was repeated twice with very similar results. Scale bars represent 100 µm. Numbers indicate raw integrated density (%), obtained from ImageJ software, compared to the maximum fluorescence level (Figure 5H).

**Figure 6:**
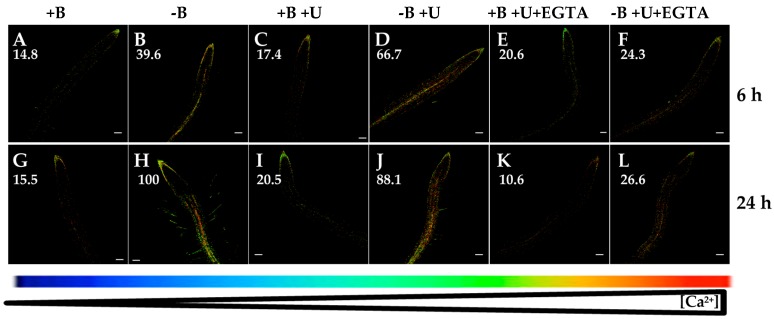
Fluorescence images of roots from *Arabidopsis* seedlings expressing the FRET-based Ca^2+^ sensor UbiQ10:YC3.6-bar#22-2 and treated or not with 1 µM U73122 and/or 1 mM EGTA. Seedlings were subjected (**B**,**D**,**F**,**H**,**J**,**L**) or not (**A**,**C**,**E**,**G**,**I**,**K**) to B deficiency for 6 (**A**–**F**) or 24 h (**G**–**L**) in the absence (**A**,**B**,**G**,**H**) or presence (**C**,**D**,**I**,**J**) of 1 µM U73122. In addition, B-sufficient and B-deficient seedlings were treated simultaneously with 1 µM U73122 and 1 mM EGTA (**E**,**F**,**K**,**L**). Fluorescence was monitored using settings for cpVenus excitation and emission. Increase in the FRET reflects higher [Ca^2+^]_cyt_ levels. For more details see Materials and Methods. Representative images: (**A**) n = 12 roots; (**B**) *n* = 12 roots; (**C**) *n* = 10 roots; (**D**) *n* = 8 roots; (**E**) *n* = 4 roots; (**F**) *n* = 5 roots; (**G**) *n* = 12 roots; (**H**) *n* = 12 roots; (**I**) *n* = 9 roots; (**J**) *n* = 10 roots; (**K**) *n* = 8 roots; and (**L**) *n* = 10 roots. Data are from a representative experiment that was repeated twice with very similar results. Scale bars represent 100 µm. Numbers indicate raw integrated density (%), obtained from ImageJ software, compared to the maximum fluorescence level (Figure 6H).

B deficiency induced an increase in the root [Ca^2+^]_cyt_ (Figure 1A,F; [30]), which was visualized as fluorescence level changes, where the root apical area did not show significant changes when *Arabidopsis* seedlings were subjected to B deprivation (Figure 1, Figure 3, Figure 4, Figure 5 and Figure 6; [30]). With the objective to determine whether this rise could be due to a calcium influx from the apoplast or intracellular organelles, fluorescence measurements in the presence of several agents affecting Ca^2+^ homeostasis were carried out. Interestingly, the membrane-impermeable calcium chelator EGTA highly reduced the increase in [Ca^2+^]_cyt_ that occurred in response to 6 and 24 h of B deprivation (Figure 3). These results pointed out that the rise in [Ca^2+^]_cyt_ induced by B deficiency would mostly be caused by calcium influx from the apoplast. Similar results were obtained in *M. domestica* pollen tube tips when these plants were subjected to B deprivation. Under this condition, a higher extracellular Ca^2+^ influx took place and brought about an increase in [Ca^2+^]_cyt_ in the pollen tube tip [31]. It was worth noting that EGTA treatment did not completely quench the fluorescence signal in 24 h-B-deficient seedlings (Figure 3H), which suggested that, even though Ca^2+^ influx from the apoplast was the main source for the rise in [Ca^2+^]_cyt_ induced by B deprivation, some influx from internal organelles was also present.

It was reported that abscisic acid (ABA) treatment induced activation of Ca^2+^ channels leading to an increase in [Ca^2+^]_cyt_ in maize and *Arabidopsis* roots [33,34,35]. Accordingly, when *Arabidopsis* seedlings were treated with ABA for 6 and 24 h, a remarkable increase in root [Ca^2+^]_cyt_ in both B-sufficient and B-deficient plants was observed (compare Figure 4A,B,G,H and Figure 4C,D,I,J). Recently, [36] have proposed a functional integration between ABA and Ca^2+^ signaling pathways, which would establish tight signaling networks rather than separate pathways. Furthermore, in *Arabidopsis* roots, [35] suggested that ABA triggers (via production of ROS) the activation of plasma membrane Ca^2+^-permeable channels, increase in [Ca^2+^]_cyt_ and, finally, inhibition of the primary root growth. Consistently with these data, *Arabidopsis* seedlings subjected to B starvation showed an increased NADPH oxidase activity and inhibition of root cell elongation [37]. In addition, diphenyleneiodonium (an inhibitor of ROS generation by NADPH oxidases) mitigated the effect of B deficiency on root cell elongation [37]. Moreover, the highest root [Ca^2+^]_cyt_ was observed in seedlings under the combined treatment of B deprivation and ABA (Figure 4D,J), as indicated by the greatest levels of fluorescence. These data would support that Ca^2+^, ABA, and ROS could be components of a signaling pathway triggered by B deficiency involved in regulating root growth [36,38].

Remarkably, in both B treatments, simultaneous application of ABA and EGTA highly reduced (Figure 4E,F,K,L) fluorescence signals compared to those from ABA treatment (Figure 4C,D,I,J). A decrease in *Arabidopsis* root [Ca^2+^]_cyt_ when ABA and EGTA were simultaneously added was also observed using the aequorin-emitted luminescence method [35]. Together, these results supported that the ABA-induced increase in [Ca^2+^]_cyt_ as well as that triggered by B deficiency were mainly a consequence of Ca^2+^ influx from the apoplast. 

Furthermore, with the aim to elucidate the involvement of some internal Ca^2+^ channels in this B-deficiency response, ruthenium red (RR), a specific compound reported to inhibit Ca^2+^ release from vacuole to cytosol, was used. RR is a membrane-permeable Ca^2+^ channel blocker that inhibits vacuolar cyclic ADP-ribose (cADPR)-dependent Ca^2+^ channels [39]. The increase in [Ca^2+^]_cyt_ under B deficiency was not decreased significantly when seedlings were treated with RR (Figure 5A–D,G–J). However, simultaneous application of RR and EGTA highly reduced the rise in [Ca^2+^]_cyt_ after 6 or 24 h of B deprivation (Figure 5F,L). This decrease in fluorescence signal was similar to that observed when EGTA was exclusively added (Figure 3D,H). Therefore, tonoplast cADPR-dependent Ca^2+^ channels did not seem to be mostly involved in the response to B starvation, and the results supported that the increase in [Ca^2+^]_cyt_ triggered by B deficiency was due mainly to Ca^2+^ influx from the apoplast.

Finally, with the purpose of ascertaining whether inositol 1,4,5-triphosphate (IP_3_)-regulated Ca^2+^ channels could be involved in the calcium response triggered by B starvation, U73122, an aminosteroid inhibitor of phospholipase C that reduces IP_3_ production and thereby inhibits the activity of these channels, was used [40,41]. U73122 application did not prevent the rise in [Ca^2+^]_cyt_ triggered by B deprivation (Figure 6A–D,G–J). Interestingly, simultaneous addition of U73122 and EGTA highly reduced the increase in [Ca^2+^]_cyt_ after 6 or 24 h of B starvation (Figure 6F,L). This effect was similar to that observed when EGTA was exclusively added (Figure 3D,H). These results seemed to suggest that phospholipase C pathway would not play an essential role in the B-starvation signaling, and that IP_3_-regulated Ca^2+^ channels would not participate in the rise of [Ca^2+^]_cyt_ associated with B deficiency. In summary, the increased root [Ca^2+^]_cyt_ in response to B deficiency was predominantly a consequence of Ca^2+^ influx from the apoplast through plasma membrane Ca^2+^ channels in an IP_3_-independent manner.

### 2.3. The Expression of Several Ca^2+^ Channel/Transporter Genes Are Altered by Compounds That Affect Ca^2+^ Homeostasis

As EGTA and ABA affected root [Ca^2+^]_cyt_ (Figure 3 and Figure 4), and B deficiency upregulated the expression of Ca^2+^ transporter genes (*CNGC19*, *ACAs* and *CAX3*) and triggered an increase in [Ca^2+^]_cyt_ (Figure 1A,F; [30]), transcriptome analyses in the presence of EGTA or ABA were performed in B-sufficient and B-deficient plants to ascertain the role these Ca^2+^ transporters could play in the regulation of root [Ca^2+^]_cyt_.

#### 2.3.1. Ethylene Glycol Tetraacetic Acid (EGTA) Treatment

**Figure 7:**
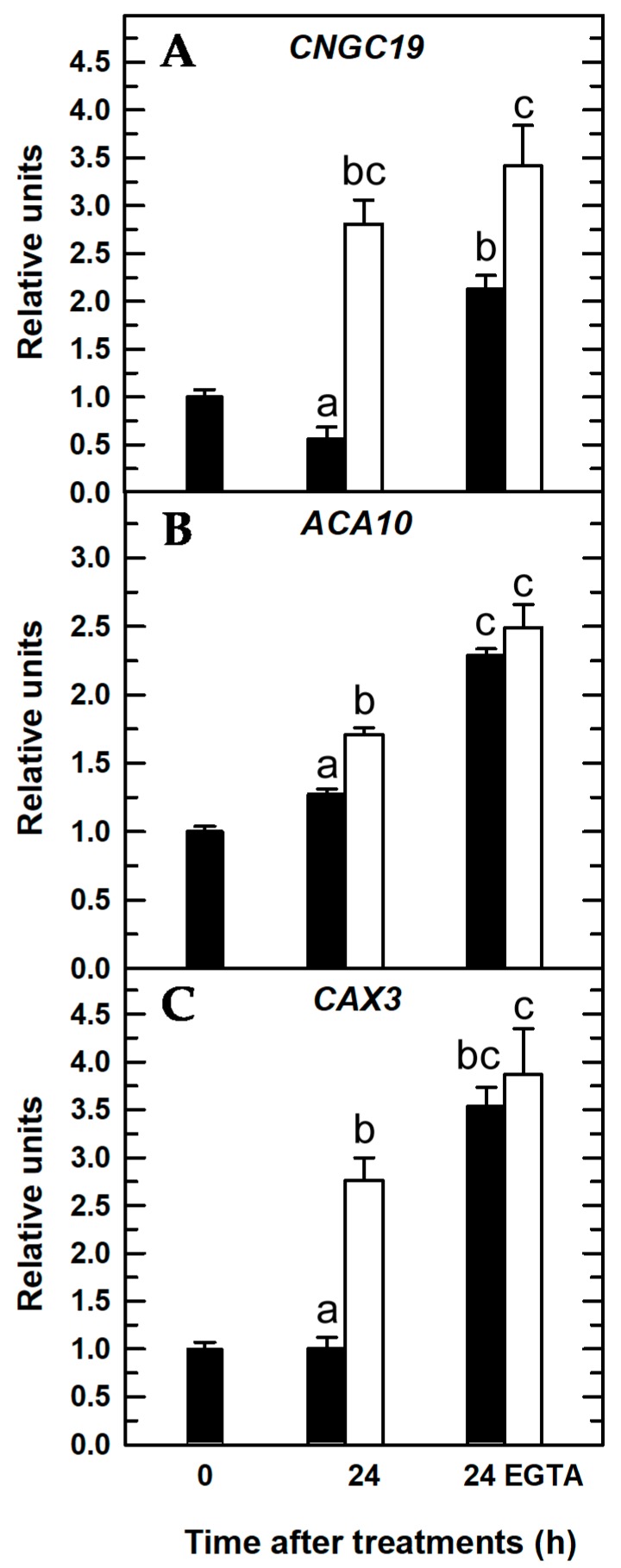
Quantitative real-time PCR analysis of transcript levels in *Arabidopsis* roots for Ca^2+^-related genes in the presence of EGTA: *CNGC19* (**A**), *ACA10* (**B**), and *CAX3* (**C**). Seedlings were subjected (open bars) or not (filled bars) to B deprivation for 24 h. For more details see Materials and Methods. The results are given as means ± SD (*n* = 4 pools of 14 separate roots). For each gene, different letters have been used to designate statistically significant differences between plants subjected or not to EGTA and B treatments. Statistical analyses were performed according to ANOVA with Tukey’s HSD test (*p* < 0.05).

**Figure 8:**
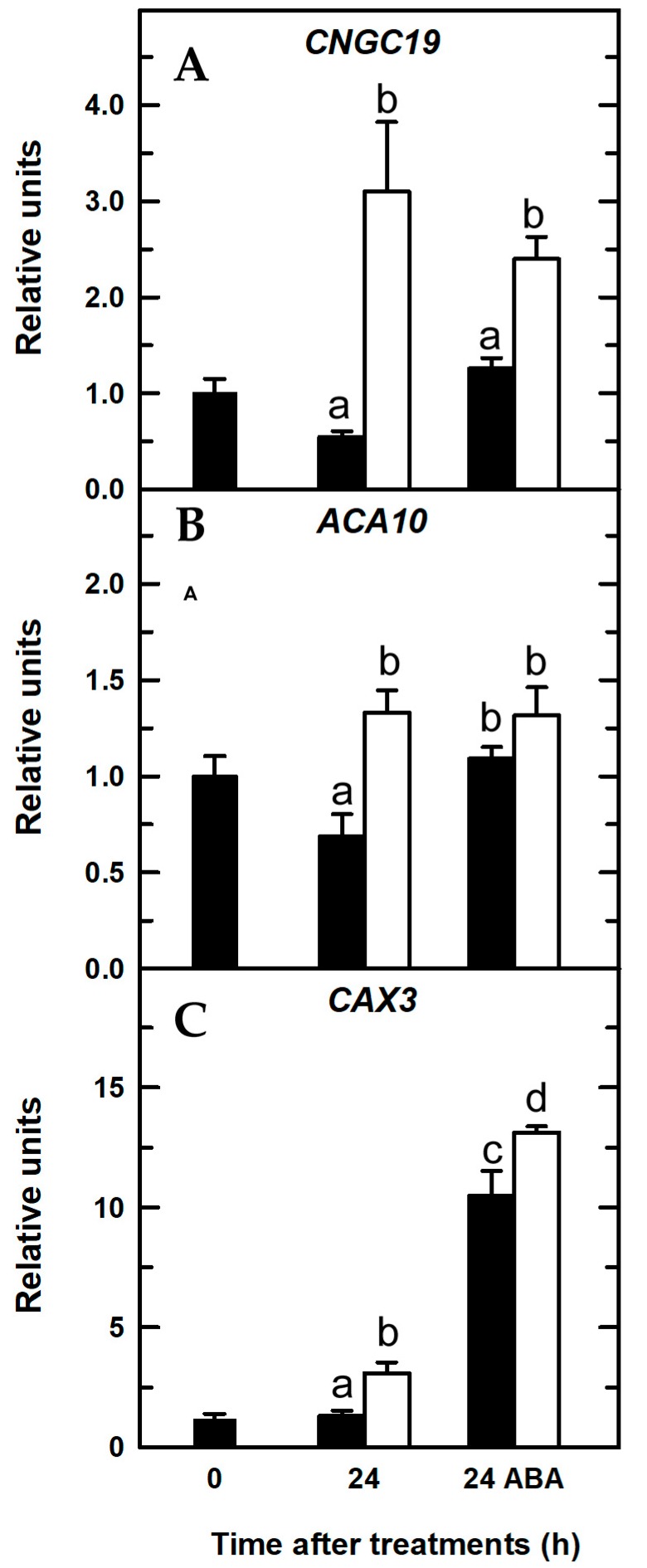
Quantitative real-time PCR analysis of transcript levels in *Arabidopsis* roots for Ca^2+^-related genes in the presence of ABA: *CNGC19* (**A**), *ACA10* (**B**), and *CAX3* (**C**). Seedlings were subjected (open bars) or not (filled bars) to B deprivation for a 24-h period. For more details see Materials and Methods. The results are given as means ± SD (*n* = 4 pools of 14 separate roots). For each gene, different letters have been used to designate statistically significant differences between plants subjected or not to ABA and B treatments. Statistical analyses were performed according to ANOVA with Tukey’s HSD test (*p* < 0.05).

As expected, root expressions of *CNGC19* (cyclic nucleotide-gated ion channel), *ACA10* (plasma membrane P_IIB_-type Ca^2+^-ATPase), and *CAX3* (vacuolar cation/proton exchanger) genes were overexpressed in *Arabidopsis* seedlings subjected to 24 h of B deficiency (Figure 7 and Figure 8; [30]). This gene overexpression was correlated with a decrease in root B concentration of B-deficient seedlings (Figure 2).

When seedlings were treated with EGTA, there was a higher expression of these three Ca^2+^-related genes irrespective of the B treatment (Figure 7), which could be explained as a general response that attempted to restore Ca^2+^ homeostasis under conditions of lower free-Ca^2+^ concentration in the apoplast owing to the presence of EGTA. In this way, the overexpression of *CNGC19* to increase [Ca^2+^]_cyt_, and *ACA10* and *CAX3* to decrease [Ca^2+^]_cyt_, would restore the Ca^2+^ electrochemical potential in roots. It was proposed that *Arabidopsis* roots responded to B deficiency by stimulating Ca^2+^ influx from the apoplast through plasma membrane CNGCs and Ca^2+^ efflux from the vacuole through CNGC19 and, thereby, increasing the [Ca^2+^]_cyt_ to trigger a Ca^2+^ signaling pathway [30,42]. Since EGTA is a membrane-impermeable Ca^2+^ chelator, its presence hinders Ca^2+^ influx from the apoplast through plasma membrane Ca^2+^ channels, such as CNGCs [5,10], and, as a result, there is lower availability of intracellular Ca^2+^ (Figure 3D,H). Very interestingly, significant differences between the *CNGC19* transcript levels of both B treatments were maintained in the presence of EGTA; the levels were significantly higher in B-deficient roots treated with EGTA (Figure 7A). In addition, under these conditions (B deficiency and EGTA) a slight fluorescence was continuously observed (Figure 3H), which suggested the involvement of CNGC19 in this response as well. These results were consistent with the previous proposal of [30,42]; our data supported that *Arabidopsis* plants responded to B deficiency, even in the presence of EGTA, by increasing *CNGC19* transcript levels to try to compensate for a lower apoplastic Ca^2+^ concentration as well as Ca^2+^ efflux from the vacuole into the cytosol through CNGC19 (Figure 7A). Accordingly, in the absence of EGTA, apoplastic free-Ca^2+^ was available for transport via plasma membrane Ca^2+^ channels, and its influx caused the most significant increase in [Ca^2+^]_cyt_ under B deprivation (Figure 3B,F), whose effect was reduced by EGTA (Figure 3D,H).

Unlike *CNGC19* gene expression, upon 24 h of EGTA treatment, no significant differences in *ACA10* and *CAX3* transcript levels were found between both B treatments (Figure 7B,C). ACA and CAX proteins removed Ca^2+^ from the cytosol to the apoplast or organelles to restore cytosolic Ca^2+^ homeostasis after exposure to several environmental stimuli [3,5,43,44]. As in the presence of EGTA, there was not a remarkable increase in [Ca^2+^]_cyt_ (Figure 3C,D,G,H) that would explain the lack of significant differences in *ACA10* and *CAX3* transcript levels between both B treatments (Figure 7B,C). Conversely, in the absence of EGTA, *ACA10* and *CAX3* gene expressions were significantly increased upon 24 h of B deprivation (Figure 7B,C); these results were consistent with those from [Ca^2+^]_cyt_ where a rise in the [Ca^2+^]_cyt_ was observed (Figure 3B,F). Therefore, *ACA10* and *CAX3* overexpression would contribute to restore [Ca^2+^]_cyt_.

#### 2.3.2. Abscisic Acid (ABA) Treatment

In vascular plants, ABA stimulates release of Ca^2+^ from intracellular stores through increased cADPR levels [45]. Moreover, in guard cells, ABA increases [Ca^2+^]_cyt_ via activation of plasmalemma Ca^2+^-permeable, nonselective cation channels and Ca^2+^ efflux from intracellular Ca^2+^ stores [46]. Consequently, a rise in the [Ca^2+^]_cyt_ was observed as early as 6 h after ABA application in both B treatments (Figure 4C,D,I,J), which was associated with increased transcript levels of the *CAX3* gene compared to those without ABA treatment (Figure 8). Interestingly, when B-deficient plants were treated with ABA, there was a clear, statistically significant increase in *CAX3* transcript abundance (Figure 8C), which was at least five times higher than that of *CNGC19* and *ACA10* genes (Figure 8A,B) and more than eight times higher when compared to the *CAX3* transcript level of B-deficient plants treated without ABA (Figure 8C). These results were consistent with those reported by [47] in cotton roots, in which the *GhCAX3* gene was upregulated by ABA treatment (*GhCAX3* is highly homologous to *AtCAX3* gene, with 74% identity and 84% similarity). ABA-induced overexpression of *CAX3* gene was especially associated with a remarkable rise in [Ca^2+^]_cyt_ observed in B-deficient seedlings treated with ABA (Figure 8C and Figure 4J, respectively). Kinetic properties of CAX transporters differed from those of ACA ones. For instance, CAX antiporters had low affinity but a high capacity for Ca^2+^ transport, whereas ACA proteins had higher affinities but a low capacity for Ca^2+^ transport [4,48]. Accordingly, it was proposed that ACA pumps were responsible for maintaining homeostasis of [Ca^2+^]_cyt_ in the resting cell, while CAX antiporters were particularly important for restoration of [Ca^2+^]_cyt_ associated with signaling pathways [44,49,50]. Therefore, increased [Ca^2+^]_cyt_ upon ABA application would trigger an overexpression of *CAX3* gene that would restore [Ca^2+^]_cyt_ to submicromolar levels. Taken together, these results suggested that CAX3 would play a major role in the restoration of Ca^2+^ homeostasis upon B starvation stimulus.

## 3. Materials and Methods

### 3.1. Plant Material and Growth Conditions

Seeds of *A. thaliana* expressing a fluorescence resonance energy transfer (FRET)-based Ca^2+^ sensor (UbiQ10:YC3.6-bar#22-2, [51]) (YC3.6) were kindly gifted by Prof. Dr. Jörg Kudla (Institut für Biologie und Biotechnologie der Pflanzen, Universität Münster, Germany). These seeds and those of the *A. thaliana* wild type (ecotype Col-0) were surface-sterilized in a 5% (v/v) hypochlorite solution for 15 min and then washed three times in ethanol and three times in sterile H_2_O. Sterile seeds were sown in square (12 cm x 12 cm) Petri dishes containing a culture medium (CM) supplemented with 2 µM H_3_BO_3_ [30] and solidified with 1% (w/v) Phytagel. After sowing, plates were cold-treated at 4 °C for 48 h in darkness to synchronize seed germination. Subsequently, plates were placed vertically in a growth chamber with 16 h light/8 h dark, 25/22 °C, 75% relative humidity, and an irradiance of 150 μmol m^−2^ s^−1^ of photosynthetically active radiation. Seedlings were grown under this condition for 5–6 d, and then sets of seedlings were transferred to fresh CM supplemented with 2 µM H_3_BO_3_ (control plants) or not (B-deficient plants). Both sets of plants were treated with or without 1 mM ethylene glycol tetraacetic acid (EGTA), 5 µM ABA, 50 µM ruthenium red (RR), or 1 µM U73122. RR is a specific chemical that inhibits Ca^2+^ release from vacuole to cytosol [39]. U73122 is an inhibitor of phospholipase C that reduces inositol 1,4,5-triphosphate (IP_3_) production [40,41].

Seedlings from each treatment were randomly harvested 0, 6, and 24 h after the onset of the experiments (zero time corresponded to 1 h after the beginning of the photoperiod), and they were used for Ca^2+^ imaging by fluorescence microscopy and gene expression measurements.

In addition, for B resupply assays, *Arabidopsis* seedlings were grown with CM supplemented with 2 µM H_3_BO_3_ for 6 d, and then seedlings were transferred to fresh CM without B for 24 h. Subsequently, plants were transferred to renewed CM but supplemented with 2 µM H_3_BO_3_ for 1, 3, 6, or 24 h. At the indicated times, images were taken by fluorescence microscopy to visualize the change in cytosolic Ca^2+^ levels.

Analytical-grade compounds were always used to prepare nutrient solutions and reagents. Purified water was obtained by a system consisting of three units (active charcoal, ion exchanger, and reverse osmosis) connected in series to an ELGA water purification system (PURELAB ultra), which supplied water with an electrical resistivity of 18.2 MΩ cm.

### 3.2. Imaging of Cytosolic Ca^2+^ Levels

Root [Ca^2+^]_cyt_ measurements were performed using an *A. thaliana* line expressing Yellow Cameleon 3.6 (YC3.6) [51]. YC3.6 structure and its fluorescence emission mechanism upon its binding Ca^2+^ were described by [51,52]. For imaging, *Arabidopsis* seedlings expressing YC3.6 were grown in CM supplemented with 2 µM H_3_BO_3_ for 5–6 d. Afterwards, plants were transferred randomly to fresh CM supplemented with (2 µM, control) or without B, and they were treated or not with EGTA, ABA, RR, or U73122 as previously described. In vivo root Ca^2+^ measurements were performed at the above indicated times after onset of the treatments on an inverted fluorescence microscope (SP5 MP, DMI6000; Leica). Excitation was provided by an argon lamp through a 458 nm filter at 30% of its intensity, and emission filters were 485/20 nm (ECFP) and 535/15 nm (cpVenus). Image acquisition was performed using LASAF (Leica), and ratio calculations and fluorescence quantifications (raw integrated density) were determined using ImageJ (http://imagej.nih.gov/ij/) software. To hold the roots in position, each seedling was submerged in CM, with (2 µM, control) or without B, and treated or not with EGTA, ABA, RR, or U73122, between a slide and a cover slip to create a sandwich to fix the root and proceed with Ca^2+^ measurements.

### 3.3. RNA Isolation, cDNA Synthesis, and Quantitative Real-Time PCR Analyses

For these determinations, *Arabidopsis* ecotype Col-0 seedlings were grown in CM supplemented with 2 µM H_3_BO_3_ for 6 d, and then they were transferred randomly to fresh CM with (2 µM, control) or without B and treated or not with EGTA or ABA, as previously described. Four pools of 14 roots from each treatment were harvested randomly 0 and 24 h after the onset of the treatments. Roots were quickly separated, dried with a paper towel, frozen in liquid nitrogen, and stored at –80 °C until further analyses.

The expression levels of *CNGC19*, *ACA10*, and *CAX3* genes were normalized to the levels of *Arabidopsis AP4M* (TAIR ID: AT4g24550), *EF1α* (TAIR ID: At1g07940), and *TON1A* (TAIR ID: At3g55000) reference genes. The following gene-specific primers were used for qRT-PCR analyses: *CNGC19* (TAIR ID: At3g17690) (forward primer CCAAGTGGCTTGGAGATACC), reverse primer TCTACCAAACCAAACATCATCATC); *ACA10* (TAIR ID: At4g29900) (forward primer AAACCGGTGGAGAAGGAACT, reverse primer CCACTAAAAGCCACCTTTGG); *CAX3* (TAIR ID: At3g51860) (forward primer TGATTCGTCATCCAAAAACG, reverse primer AAGCTCCCTCCCTCATTCAT); *AP4M* (TAIR ID: AT4g24550) (forward primer AGCATACAC TGCGTGCAAAG, reverse primer TCGCCTGTGTCACATATCTC); *EF1α* (TAIR ID: At1g07940) (forward primer CCTTGGTGTCAAGCAGATGA, reverse primer TGAAGACACCTCCTTGATGATTT); and *TON1A* (TAIR ID: At3g55000) (forward primer: TGTGAGGGATGGAACAAATG; reverse primer: AACGCAGTTGCAAATAAAGGA). *CNGC19*, *ACA10*, and *CAX3* gene expressions were analyzed using the geometric mean of the three housekeeping genes above mentioned, as reported by [53]. Efficiency of qRT-PCR reactions was higher than 94%.

### 3.4. Total Boron Content Analyses

Pools of frozen roots were ground to a fine powder in a mortar precooled with liquid nitrogen, transferred to porcelain crucibles, and dried out at 80 °C for 72 h. Subsequently, dried pools were weighed and burnt to ashes at 550 °C for 6 h. Ashes, once at room temperature within a desiccator, were dissolved with 0.1 M HCl, and then B was determined following the azomethine-H method as described by [54].

### 3.5. Statistical Analysis

The data shown were mean values ±SD. Results were statistically analyzed using one-way analysis of variance (ANOVA). Differences among treatment means were evaluated using Tukey’s honestly significant difference test (*p* < 0.05). Regarding Ca^2+^ imaging by fluorescence microscopy, representative images from 4 to 13 primary roots for each treatment were shown. Data were from a representative experiment that was repeated twice with very similar results. 

## 4. Conclusions

In summary, it can be concluded that B deficiency elicits increased [Ca^2+^]_cyt_ after 6 and 24 h of this nutrient stress, which is due mainly to Ca^2+^ influx across the plasma membrane from the apoplast, even though it cannot be ruled out that Ca^2+^ also comes from the vacuole through the tonoplast CNGC19 channel. When B-sufficient conditions are re-established, [Ca^2+^]_cyt_ is gradually restored. CAX3 would play a major role in the restoration of Ca^2+^ homeostasis after 24 h of B deficiency.

## Abbreviations

ABA	Abscisic Acid
ACA	Autoinhibited Ca^2+^-ATPases
cADPR	Cyclic ADP-ribose
CAX	Cation/H^+^ Exchanger
[Ca^2+^]_cyt_	Cytosolic Calcium Concentration
CM	Culture Medium
CNGC	Cyclic Nucleotide-Gated Ion Channels
EGTA	Ethylene Glycol Tetraacetic Acid
IP_3_	Inositol 1,4,5-Triphosphate
FRET	Fluorescence Resonance Energy Transfer
ROS	Reactive Oxygen Species
RR	Ruthenium Red
qRT-PCR	Quantitative Real Time-PCR

## References

[1] López-Bucio J., Cruz-Ramírez A., Herrera-Estrella L. (2003). The role of nutrient availability in regulating root architecture. Curr. Opin. Plant Biol..

[2] Kochian L.V., Lucas W.J. (2014). Plant mineral nutrient sensing and signaling. J. Integr. Plant Biol..

[3] Dodd A.N., Kudla J., Sanders D. (2010). The language of calcium signaling. Annu. Rev. Plant Biol..

[4] Conn S., Gilliham M. (2010). Comparative physiology of elemental distributions in plants. Ann. Bot..

[5] Sanders D., Pelloux J., Brownlee C., Harper J.F. (2002). Calcium at the crossroads of signaling. Plant Cell.

[6] Hetherington A.M., Brownlee C. (2004). The generation of Ca^2+^ signals in plants. Annu. Rev. Plant Biol..

[7] González-Fontes A., Navarro-Gochicoa M.T., Ceacero C.J., Herrera-Rodríguez M.B., Camacho-Cristóbal J.J., Rexach J., Hossain M.A., Kamiya T., Burritt D.J., Phan Tran L.S.P., Fujiwara T. (2017). Understanding calcium transport and signaling, and its use efficiency in vascular plants. Plant Macronutrient Use Efficiency: Molecular and Genomic Perspectives in Crop Plants.

[8] Swarbreck S.M., Colaço R., Davies J.M. (2013). Plant calcium-permeable channels. Plant Physiol..

[9] Finka A., Cuendet A.F.H., Maathuis F.J.M., Saidi Y., Goloubinoff P. (2012). Plasma membrane cyclic nucleotide gated calcium channels control land plant thermal sensing and acquired thermotolerance. Plant Cell.

[10] DeFalco T.A., Moeder W., Yoshioka K. (2016). Opening the gates: Insights into cyclic nucleotide-gated channel-mediated signaling. Trends Plant Sci..

[11] Yuen C.C.Y., Christopher D.A. (2013). The group IV-A cyclic nucleotide-gated channels, CNGC19 and CNGC20, localize to the vacuole membrane in *Arabidopsis thaliana*. AoB Plants.

[12] Pittman J.K., Bonza M.C., De Michelis M.I., Geisler M., Venema K. (2011). Ca^2+^ pumps and Ca^2+^ antiporters in plant development. Transporters and Pumps in Plant Signaling.

[13] Warington K. (1923). The effect of boric acid and borax on the broad bean and certain other plants. Ann. Bot..

[14] Goldbach H.E., Wimmer M. (2007). Boron in plants and animals: Is there a role beyond cell-wall structure?. J. Plant Nutr. Soil Sci..

[15] Tanaka M., Fujiwara T. (2008). Physiological roles and transport mechanisms of boron: Perspectives from plants. Eur. J. Physiol..

[16] Shorrocks V.M. (1997). The occurrence and correction of boron deficiency. Plant Soil.

[17] Herrera-Rodríguez M.B., González-Fontes A., Rexach J., Camacho-Cristóbal J.J., Maldonado J.M., Navarro-Gochicoa M.T. (2010). Role of boron in vascular plants and response mechanisms to boron stress. Plant Stress.

[18] Camacho-Cristóbal J.J., Navarro-Gochicoa M.T., Rexach J., González-Fontes A., Herrera-Rodríguez M.B., Hossain M.A., Kamiya T., Burritt D.J., Phan Tran L.S., Fujiwara T. (2018). Plant response to boron deficiency and boron use efficiency in crop plants. Plant Micronutrient Use Efficiency: Molecular and Genomic Perspectives in Crop Plants.

[19] Kobayashi M., Matoh T., Azuma J. (1996). Two chains of rhamnogalacturonan II are cross-linked by borate-diol ester bonds in higher plant cell walls. Plant Physiol..

[20] O’Neill M.A., Warrenfeltz D., Kates K., Pellerin P., Doco T., Darvill A.G., Albersheim P. (1996). Rhamnogalacturonan-II, a pectic polysaccharide in the walls of growing plant cell, forms a dimer that is covalently cross-linked by a borate ester. J. Biol. Chem..

[21] Blevins D.G., Lukaszewski K.M. (1998). Boron in plant structure and function. Annu. Rev. Plant Physiol. Plant Mol. Biol..

[22] Brown P.H., Bellaloui N., Wimmer M.A., Bassil E.S., Ruiz J., Hu H., Pfeffer H., Dannel F., Römheld V. (2002). Boron in plant biology. Plant Biol..

[23] Bolaños L., Lukaszewski K., Bonilla I., Blevins D. (2004). Why boron?. Plant Physiol. Biochem..

[24] Camacho-Cristóbal J.J., Rexach J., González-Fontes A. (2008). Boron in plants: Deficiency and toxicity. J. Integr. Plant Biol..

[25] Camacho-Cristóbal J.J., Rexach J., Herrera-Rodríguez M.B., Navarro-Gochicoa M.T., González-Fontes A. (2011). Boron deficiency and transcript level changes. Plant Sci..

[26] Reid R. (2014). Understanding the boron transport network in plants. Plant Soil.

[27] Hua Y., Feng Y., Zhou T., Xu F. (2017). Genome-scale mRNA transcriptomic insights into the responses of oilseed rape (*Brassica napus* L.) to varying boron availabilities. Plant Soil.

[28] Liu X., Zhang J.-W., Guo L.-X., Liu Y.-Z., Jin L.-F., Hussain S.B., Du W., Deng Z., Peng S.-A. (2017). Transcriptome changes associated with boron deficiency in leaves of two citrus scion-rootstock combinations. Front. Plant Sci..

[29] Koshiba T., Kobayashi M., Ishihara A., Matoh T. (2010). Boron nutrition of cultured tobacco BY-2 cells. VI. Calcium is involved in early responses to boron deprivation. Plant Cell Physiol..

[30] Quiles-Pando C., Rexach J., Navarro-Gochicoa M.T., Camacho-Cristóbal J.J., Herrera-Rodríguez M.B., González-Fontes A. (2013). Boron deficiency increases the levels of cytosolic Ca^2+^ and expression of Ca^2+^-related genes in *Arabidopsis thaliana* roots. Plant Physiol. Biochem..

[31] Fang K.F., Du B.S., Zhang Q., Xing Y., Cao Q.Q., Qin L. (2019). Boron deficiency alters cytosolic Ca^2+^ concentration and affects the cell wall components of pollen tubes in *Malus domestica*. Plant Biol..

[32] Gilroy S., Suzuki N., Miller G., Choi W.G., Toyota M., Devireddy A.R., Mittler R. (2014). A tidal wave of signals: Calcium and ROS at the forefront of rapid systemic signaling. Trends Plant Sci..

[33] White P.J., Bowen H.C., Demidchik V., Nichols C., Davies J.M. (2002). Genes for calcium-permeable channels in the plasma membrane of plant root cells. Biochim. Biophys. Acta.

[34] Bai L., Zhang G., Zhou Y., Zhang Z., Wang W., Du Y., Wu Z., Song C.-P. (2009). Plasma membrane-associated proline-rich extensin-like receptor kinase 4, a novel regulator of Ca^2+^ signalling, is required for abscisic acid responses in *Arabidopsis thaliana*. Plant J..

[35] Jiao Y., Sun L., Song Y., Wang L., Liu L., Zhang L., Liu B., Li N., Miao C., Hao F. (2013). AtrbohD and AtrbohF positively regulate abscisic acid-inhibited primary root growth by affecting Ca^2+^ signalling and auxin response of roots in Arabidopsis. J. Exp. Bot..

[36] Edel K.H., Kudla J. (2016). Integration of calcium and ABA signaling. Curr. Opin. Plant Biol..

[37] Camacho-Cristóbal J.J., Martín-Rejano E.M., Herrera-Rodríguez M.B., Navarro-Gochicoa M.T., Rexach J., González-Fontes A. (2015). Boron deficiency inhibits root cell elongation via an ethylene/auxin/ROS-dependent pathway in Arabidopsis seedlings. J. Exp. Bot..

[38] González-Fontes A., Herrera-Rodríguez M.B., Martín-Rejano E.M., Navarro-Gochicoa M.T., Rexach J., Camacho-Cristóbal J.J. (2016). Root responses to boron deficiency mediated by ethylene. Front. Plant Sci..

[39] Muir S.R., Bewell M.A., Sanders D., Allen G.J. (1997). Ligand-gated Ca^2+^ channels and Ca^2+^ signalling in higher plants. J. Exp. Bot..

[40] Franklin-Tong V.E., Drobak B.K., Allan A.C., Watkins P.A.C., Trewavas A.J. (1996). Growth of pollen tubes of *Papaver rhoeas* is regulated by a slow-moving calcium wave propagated by inositol 1,4,5-triphosphate. Plant Cell.

[41] Staxén I., Pical C., Montgomery L.T., Gray J.E., Hetherington A.M., McAinsh M.R. (1999). Abscisic acid induces oscillations in guard-cell cytosolic free calcium that involve phosphoinositide-specific phospholipase C. Proc. Natl. Acad. Sci. USA.

[42] González-Fontes A., Navarro-Gochicoa M.T., Camacho-Cristóbal J.J., Herrera-Rodríguez M.B., Quiles-Pando C., Rexach J. (2014). Is Ca^2+^ involved in the signal transduction pathway of boron deficiency? New hypotheses for sensing boron deprivation. Plant Sci..

[43] Kudla J., Batistic O., Hashimoto K. (2010). Calcium signals: The lead currency of plant information processing. Plant Cell.

[44] Bose J., Pottosin I.I., Shabala S.S., Palmgren M.G., Shabala S. (2011). Calcium efflux systems in stress signaling and adaptation in plants. Front. Plant Sci..

[45] Wu Y., Kuzma J., Maréchal E., Graeff R., Lee H.C., Foster R., Chua N.-H. (1997). Abscisic acid signaling through cyclic ADP-ribose in plants. Science.

[46] Munemasa S., Hauser F., Park J., Waadt R., Brandt B., Schroeder J.I. (2015). Mechanisms of abscisic acid-mediated control of stomatal aperture. Curr. Opin. Plant Biol..

[47] Xu L., Zahid K.R., He L., Zhang W., He X., Zhang X., Yang X., Zhu L. (2013). *GhCAX3* gene, a novel Ca^2+^/H^+^ exchanger from cotton, confers regulation of cold response and ABA induced signal transduction. PLoS ONE.

[48] Hirschi K. (2001). Vacuolar H^+^/Ca^2+^ transport: Who’s directing the traffic?. Trends Plant Sci..

[49] Sze H., Liang F., Hwang I., Curran A.C., Harper J.F. (2000). Diversity and regulation of plant Ca^2+^ pumps: Insights from expression in yeast. Annu. Rev. Plant Physiol. Plant Mol. Biol..

[50] Pittman J.K., Hirschi K.D. (2016). CAX-ing a wide net: Cation/H^+^ transporters in metal remediation and abiotic stress signaling. Plant Biol..

[51] Krebs M., Held K., Binder A., Hashimoto K., Herder G.D., Parniske M., Kudla J., Schumacher K. (2012). FRET-based genetically encoded sensors allow high-resolution live cell imaging of Ca^2+^ dynamics. Plant J..

[52] Miyawaki A., Llopis J., Heim R., McCaffery J.M., Adams J.A., Ikura M., Tsien R.Y. (1997). Fluorescent indicators for Ca^2+^ based on green fluorescent proteins and calmodulin. Nature.

[53] Vandesompele J., De Preter K., Pattyn F., Poppe B., Van Roy N., De Paepe A., Speleman F. (2002). Accurate normalization of real-time quantitative RT-PCR data by geometric averaging of multiple internal control genes. Genome Biol..

[54] Beato V.M., Rexach J., Navarro-Gochicoa M.T., Camacho-Cristóbal J.J., Herrera-Rodríguez M.B., González-Fontes A. (2010). A tobacco asparagine synthetase gene responds to carbon and nitrogen status and its root expression is affected under boron stress. Plant Sci..

